# User Experience Testing of the Meta Quest 2 for Integration With the Virtual Reality Simulation for Dementia Coaching, Advocacy, Respite, Education, Relationship, and Simulation (VR-SIM CARERS) Program

**DOI:** 10.7759/cureus.66314

**Published:** 2024-08-06

**Authors:** Emily O'Hara, Refka Al-Bayati, Mary Chiu, Adam Dubrowski

**Affiliations:** 1 Health Sciences, Ontario Tech University, Oshawa, CAN; 2 Toronto Dementia Research Alliance, Ontario Tech University, Ontario Shores Centre for Mental Health Sciences, Toronto, CAN; 3 MaxSIMhealth Group, Ontario Tech University, Oshawa, CAN

**Keywords:** dementia caregivers, dementia care, dementia, simulation, virtual reality

## Abstract

Caregivers (CGs) of persons with dementia (PWDs) face numerous challenges, including learning about the condition, managing behavioral symptoms, and prioritizing their own well-being. Virtual reality (VR) technology has emerged as a promising tool to adopt certain elements of existing CG psychoeducation programs, such as the Reitman Centre CARERS (coaching, advocacy, respite, education, relationship, and simulation) program, which has been shown effective in reducing CG burden and stress and building the required skills for caring for PWD. Recently, we have developed a VR prototype utilizing Meta Quest 2 (Meta, Menlo Park, CA, USA), which will be referred to as the (virtual reality simulation for dementia CARERS) VR-SIM CARERS program. This technical report aims to describe the early stages of intervention modeling by testing user experiences related to the hardware used. The Meta Quest 2 VR system is chosen for its accessibility and functionality, aiming to ensure widespread access. Through interviews and observational techniques, we explored CGs age-matched controls’ attitudes, comfort, and proficiency with the Meta Quest 2 VR system, which are crucial for informing technological choices. Initial findings revealed mixed attitudes, comfort, and proficiency about the Meta Quest 2 VR system. Although further testing of the Meta Quest 2 VR system within the CG community is warranted, the interpretation of these preliminary results indicates that the VR-SIM CARERS program should have minimal technological skill requirements for user engagement or provide in-depth training resources for the CGs who choose to use the system.

## Introduction

Dementia presents a complex and pressing public health challenge. Dementia is an acquired condition where an individual experiences significant declines in cognitive abilities, impacting their social and occupational functions [1]. Due to the unique nature of the disease, behavioral and psychological symptoms of dementia significantly impact the quality of life of both the person with dementia (PWD) and their caregiver (CG) [2]. Caring for a PWD requires CGs to learn a variety of new skills, including managing social interactions, learning about the disease, and physically caring for the PWD, all while prioritizing their own physical and mental well-being [3]. In addition, CGs are often required to take the PWD to appointments and, at times, to support groups to help them develop their caregiving skills and manage their mental health.

Regarding CG training for PWD, the Reitman Centre (coaching, advocacy, respite, education, relationship, and simulation) CARERS program stands out as an evidence-supported psychotherapeutic intervention [4]. The in-person program was found to effectively improve CG burden and competence and reduce stress for individuals caring for PWD [4]. It presents an opportunity to adopt simulation-based experiential learning into a self-directed learning program, specifically through a virtual reality (VR) environment. VR technology constructs a 3D virtual realm where users can engage and interact [5]. Consisting of a wearable headset and controllers, a VR program generates immersive visual and auditory experiences, capable of replicating realistic settings [5]. This versatile technology offers numerous practical applications, including skills-building for dementia CGs.

The successful implementation of the virtual reality simulation for dementia CARERS (VR-SIM CARERS) program relies on several key factors. Among these, three critical elements stand out: the willingness of CGs to embrace the hardware associated with VR, their acceptance of the software used in conjunction with the hardware, and the degree of realism achieved in the scenarios designed for deployment within this technological framework. According to the adapted Medical Research Framework for the development of complex simulation-based interventions [6], this initial step is a critical part of intervention modeling.

This technical report primarily focuses on exploring and elucidating the first of these factors: the acceptance of the hardware component. Understanding how CGs perceive and interact with VR equipment is crucial for determining the feasibility and effectiveness of incorporating VR technology into the CARERS program.

To accomplish this, we conducted an assessment of participants' attitudes and experiences with VR hardware. Through interviews and observational techniques, we sought to gain insights into their comfort level, proficiency, and willingness to utilize VR devices.

## Technical report

Context

The CARERS program is a comprehensive initiative designed to support and empower CGs in their crucial roles of caring for aging or disabled loved ones. Through a multi-faceted approach, the program offers a range of services and resources to address the diverse needs and challenges CGs face. Coaching components provide personalized guidance and support to CGs, helping them navigate the complexities of caregiving and develop effective coping strategies. Advocacy efforts seek to raise awareness of CGs' rights and needs while advocating for policy changes and resources to better support their caregiving responsibilities. Respite services offer CGs much-needed breaks from their caregiving duties, allowing them to recharge and attend to their well-being. Education initiatives provide CGs with valuable information and training on topics such as caregiving techniques, health management, and self-care practices. Relationship-building activities foster a sense of community and connection among CGs, offering opportunities for mutual support and shared experiences. Lastly, simulation activities utilize innovative technology, such as VR, to provide CGs with realistic scenarios and training opportunities to enhance their caregiving skills and confidence. Together, these components form a holistic and comprehensive support system for CGs, helping them navigate the challenges of caregiving while promoting their well-being and the quality of care for their loved ones.

In Canada, CGs of PWDs span a wide range of age brackets, but certain age groups are more prevalent within this demographic. Middle-aged adults, typically aged between 40 and 60, make up a significant proportion of CGs, often referred to as the "sandwich generation," as they juggle caregiving responsibilities for aging parents with their own family and work obligations [6]. Additionally, older adults aged 60 and above also play a prominent role in dementia caregiving, as they may be caring for spouses or siblings with dementia while also dealing with their age-related health issues [7]. It's also important to note that some younger adults, particularly those in their 20s and 30s, may also serve as CGs for parents or grandparents with dementia. However, they may face unique challenges balancing caregiving with other life responsibilities and career aspirations. Overall, CGs of PWDs in Canada come from diverse age groups, each facing distinct challenges and experiences associated with their stage of life and caregiving role.

Adults may encounter various challenges when using VR hardware due to factors such as their competencies and preferences [8]. One potential difficulty is the phenomenon of simulator sickness, characterized by symptoms such as nausea, dizziness, and disorientation, which can arise from mismatches between visual and vestibular cues during VR experiences [9]. However, what is most important to the aims of this technical report are issues related to ergonomics and comfort, such as the weight and fit of VR headsets, which may pose challenges for individuals with physical limitations or musculoskeletal disorders [10]. To better understand and address these difficulties, theoretical frameworks such as cognitive load theory can be applied to examine how the cognitive demands of interacting with VR technology may exceed individuals' cognitive capacities, leading to performance decrements and increased mental workload [11]. By considering factors such as age-related cognitive decline and cognitive load, designers and developers can make more informed decisions related to choices of VR technologies or additional training requirements to better accommodate the needs and capabilities of adults in the 40-60 age range, thereby enhancing usability and user experience.

Input

**Figure 1:**
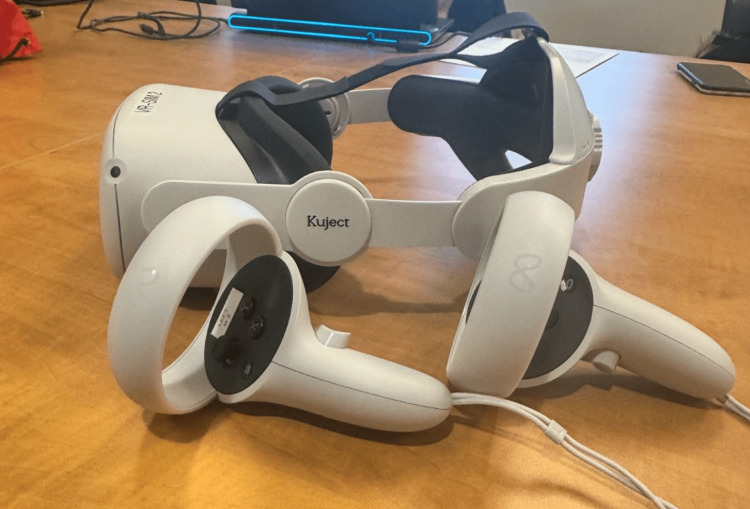
Meta Quest 2 headset and controller used in the testing described in this technical report

The Meta Quest 2 (Meta, Menlo Park, CA, USA) serves as the preferred VR system for the VR-SIM CARERS program due to its functionality and accessibility (Figure 1) [12]. The Meta Quest 2 offers a consumer-grade VR experience, ensuring widespread availability for users to access and participate in VR activities. Sufficient accessibility to VR headsets is crucial for the project, as the end goal is to create a VR simulation environment accessible to all CGs of PWDs.

Operating through a VR headset with built-in audio, the Meta Quest 2 is designed to accommodate users of all types. The unit features adjustable lenses that accommodate various eye sizes, ensuring ideal visuals for all users. Additionally, the flexible head strap guarantees a comfortable and secure fit regardless of head size. Equipped with two touch controllers, the Meta Quest 2 allows users to interact seamlessly with the virtual environment. These controllers feature sensors that track the user's position in physical space, along with buttons and joysticks for intuitive interaction within the virtual realm.

Process

The participants in this study consisted of adults in the Durham Region area. The inclusion criteria for participation included being 18 years of age or older, being able to provide informed consent, and being able to read and speak English. Eight participants were recruited for this study.

Prior to data collection, ethical approval was obtained from the Ontario Tech University Research Ethics Board (approval number: 22-031-B). Participants who expressed interest in participating were provided with detailed information about the study objectives, procedures, and potential risks and benefits, and written informed consent was obtained from each participant.

Data was collected during two technology roadshows hosted at Ontario Tech University in Oshawa, Ontario, and at the Ontario Shores Centre for Mental Health Sciences in Whitby, Ontario. Roadshows are mobile user experience testing events consisting of the process outlined below. Data was collected using a Think Aloud (TAO) protocol, which involves participants openly expressing their thoughts and feelings while performing a specific task [13], in this case, interacting with the Oculus Tutorial App using a Meta Quest 2. The roadshows were conducted in a semi-structured interview format. By using TAO and the semi-structured interview format, our goal was to create an environment where the participant feels comfortable enough to be completely transparent and tell us their unfiltered thoughts about Meta Quest 2 while using the system, including their comfortability, any pain points or areas of difficulty, preferences, and their honest opinion about integrating Meta Quest 2 in the VR-SIM CARERS program.

The testing protocol consisted of the following steps:

Introduction and Orientation

Research personnel gave a brief presentation about the VR-SIMS CARERS program and an overview of the Meta Quest 2, including instructions on wearing the VR headset. Participants were introduced to the TAO process and were briefed on the tasks they would be asked to perform during the TAO session.

Task Instructions

Participants were given clear, step-by-step auditory and visual instructions within the Oculus Tutorial App, the application used for user experience testing. The instructions provided guidance on using the VR controllers, navigating within the VR environment, and interacting with objects, such as picking items up and releasing them. Within the Oculus Tutorial App, participants were asked to press different buttons on each controller, pick up and stack blocks, and fly a paper airplane.

Thinking Aloud

Participants were instructed to “think aloud” as they engaged with the VR technology, verbalizing their thoughts, observations, and reactions in real time in regard to the ease of use of this technology. Participants were encouraged to express any difficulties, challenges, or concerns they encountered while using the VR hardware without censoring or filtering their feedback [13].

Observational Notes

Researchers observed participants' actions during the TAO session to supplement verbal feedback. Detailed notes were taken on usability issues, technical glitches, and participants' emotional responses or physical discomfort while interacting with the VR hardware.

Debriefing and Feedback

Following the TAO session, a debriefing discussion was conducted with participants to gather additional insights and reflections on their experiences with the VR hardware. Participants were invited to share any suggestions, preferences, or concerns regarding the design, functionality, or usability of the VR technology.

Qualitative data collected during the TAO sessions, including verbal feedback and observational notes, was analyzed using thematic analysis [14] to identify recurring patterns, themes, and insights related to the acceptability of VR technology hardware among CGs of PWDs. Themes were derived inductively from the raw data, with consensus reached through iterative discussions among the research team through the use of Google Docs (Google LLC, California, USA) and in-person brainstorming sessions.

Products

**Figure 2:**
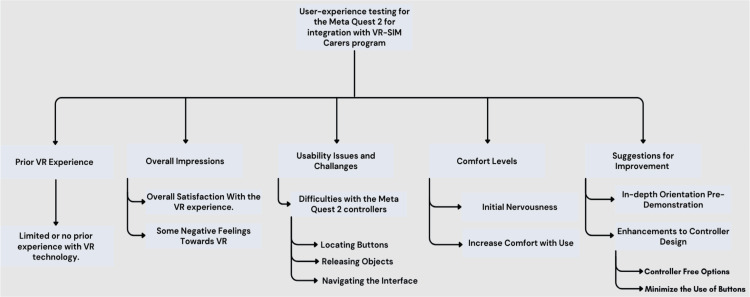
User experience testing for the Meta Quest 2 for integration with the VR-SIM CARERS program VR-SIM CARERS program: virtual reality simulation for dementia coaching, advocacy, respite, education, relationship, and simulation program

The analysis of participant feedback regarding the usability of Meta Quest 2 revealed several vital observations and trends among participants. The observations provided insight into common experiences and challenges encountered by participants. Observations were grouped into the following themes, as explained below and in Figure 2. Participant answers to the TAO questions are detailed in Table 1.

**Table 1:** TAO questions for user experience testing of Meta Quest 2 integration with the VR-SIM CARERS program TAO: Think Aloud, VR-SIM CARERS program: virtual reality simulation for dementia coaching, advocacy, respite, education, relationship, and simulation program

	Participant A	Participant B	Participant C	Participant D	Participant E	Participant F	Participant G	Participant H
Before simulation								
Has the participant used VR before?	No	No	Yes	No	No	No	No	No
Is the participant nervous about completing the simulation?	Question not asked	Question not asked	No	No	No	No	No	Mildly nervous
During simulation								
Is the participant comfortable in the seated position?	Question not asked	Question not asked	Yes	Yes	Yes	Question not asked	Yes	Yes
Is the participant comfortable handling the controllers?	Question not asked	Question not asked	Yes	Yes	The participant had minor difficulties handling the controllers	Question not asked	Yes	Yes
Does the participant feel immersed in VR?	The participant felt immersed in the VR	Question not asked	Yes	Yes	Question not asked	Question not asked	Yes	Yes
Does the participant find the controllers to be natural?	No	No	No	No	No	Question not asked	Yes	No
After simulation								
What was the participants general experience with the VR simulator?	Participant said the experience was interesting	Question not asked	Participant said the experience was good	Participant said the experience was good	Participant said the experience was interesting	The participant said the experience was fun once they were familiar with the controls	The participant found the experience to be confusing	Participant said the experience was good
How would the participant improve the VR experience?	Question not asked	Question not asked	The participant suggested improving the controllers as the button were hard to locate	The participant suggested improving the controllers and said that a glove-like controller would be easier for them to use	The participant suggested making the headset lighter as it felt heavy to them	Question not asked	Question not asked	The participant had no suggestions
Does the participant think doing the simulation a second time would make them more comfortable with the controls?	The participant would feel more comfortable with a more robust orientation of VR	Question not asked	The participant would feel more comfortable doing the simulation a second time	The participant would feel more comfortable doing the simulation a second time	The participant believes they would feel more comfortable doing the simulation a second time	Question not asked	Question not asked	Question not asked

Prior Experience

The data revealed that participants generally had limited prior experience with VR technology, indicating a predominantly novice user demographic. During an orientation session, a participant noted, “No, (I) have not used VR before” (participant A).

Overall Impressions

Feedback regarding enjoyment of Meta Quest 2 was mixed. While most participants expressed satisfaction and enjoyment with the experience, some individuals reported negative feelings toward it. This variance in feedback emphasizes the subjective nature of user experiences in VR environments and with VR technology, highlighting the importance of catering to diverse user preferences and needs. The majority of participants noted that they believed they would be more comfortable if they completed this roadshow event a second time. When asked if they thought they would be more comfortable trying VR a second time, one participant said, “Of course, (it) was getting easier the more (I) did it” (participant D).

Usability Issues and Challenges

Many participants encountered difficulties with the Meta Quest 2 controllers, highlighting usability concerns. Specifically, challenges were noted in locating physical buttons on the controller, releasing virtual objects, and navigating the interface. One participant noted that the “buttons are too close” (participant E). This feedback underscores the need for potential improvements in controller design to enhance usability.

Positive Feedback and Satisfaction

While the feedback regarding enjoyment of Meta Quest 2 was mixed, multiple participants expressed satisfaction. For example, after completing a VR event, a participant noted that “it was fun” (participant E). Another participant expressed that they “enjoyed the VR visuals and colors” (participant F).

Comfort Levels

As indicated above, the majority of participants had limited experience with the use of VR technology. Prior to the roadshow event, participants were asked if they were nervous about completing the activity, and the majority of the participants responded that they were not. During the event, most participants noted being comfortable in the seated position.

Suggestions for Improvements

After each user experience testing session, a debriefing session was conducted, which allowed the participants to provide us with valuable feedback and make suggestions. One participant noted that “a more robust orientation would be helpful to learn how to operate (the VR system)” (participant A). Another participant suggested that a “glove type (controller) would be more intuitive than the buttons (as) it would be natural” (participant D).

## Discussion

The aim of the user experience testing of the Meta Quest 2 was to determine its suitability for simulating dementia care training and specifically integrate its use in the VR-SIM CARERS program.

The use of the TAO method enabled the collection of valuable feedback from participants, providing insight into the usability of the device and its effectiveness in creating an immersive training space. The data additionally underscored struggles with the VR controllers and reported mixed feelings of enjoyment about using the VR system. The analysis of this data highlights areas of strength in Meta Quest 2 but also identifies areas for potential improvement. These improvements could be made by either adding components that utilize a constraint-based approach [15] or by providing additional training resources before full-scale implementation [16].

The feedback provided by numerous participants during the TAO sessions expressing their dislikes, such as their enjoyment of the experience and their struggles with the controllers, suggests that we successfully created an open and transparent environment where individuals felt comfortable sharing their honest opinions. The TAO protocol elicited candid feedback, indicating our readiness to proceed with the protocol without any further modifications.

Most participants demonstrated acceptance of Meta Quest 2 following the TAO sessions, despite reporting challenges with system usage. This indicates that participants are willing to adapt to the technology, which is further highlighted in their expressed interest in trying the simulation a second time. This suggests that participants were engaged with the VR experience, which is crucial for the successful implementation of VR-SIM CARERS. Notably, most difficulties reported by participants were struggles with the controllers, signifying an area of potential improvement. Additionally, the seated position was widely recognized as comfortable, underscoring its significant contribution to a positive user experience. This finding is in line with a phenomenon often referred to as the "technology acceptance paradox" [17]. This paradox arises when individuals perceive barriers or difficulties in using technology, yet they still choose to adopt it because they recognize the potential benefits or advantages it offers. That is, in the case of Meta Quest 2, despite facing technological challenges, individuals who were briefed to empathize with CGs' struggle and need to engage in the VR-SIM CARERS, may be willing to overlook the technological barriers if they believe that the benefits outweigh the costs or difficulties associated with using the technology.

The participants also suggested several solutions, aiming at improving interactivity, leading to enhanced enjoyment and a reduction in struggles associated with VR technology. As suggested by a participant, the use of glove-shaped controllers, also known as VR haptic gloves, could indicate a promising avenue for enhancing interactions in the virtual environment. A VR haptic glove is worn by users with sensors to track hand movements [18]. The VR haptic glove would replace the controller and allow participants to use their hands, reducing the learning curve associated with the Meta Quest 2 controller. However, VR haptic gloves are not widely available on the consumer market, suggesting feasibility issues with integration into the VR-SIM CARERS program. Alternative options include control-free VR systems. Several such systems exist on the market, all of which have integrated controller-free interactions, allowing users to engage with virtual environments through gestures, body movements, or other natural interactions without the need for handheld controllers. One notable example is the Oculus Quest/Quest 2, a standalone VR headset equipped with hand-tracking technology that enables users to interact with virtual content using their hands and fingers [12]. Similarly, the Microsoft HoloLens (Microsoft Corporation, Redmond, WA, USA), although primarily an augmented reality device, supports hand-tracking capabilities, allowing users to manipulate holographic objects and engage with virtual content through gestures. Additionally, Leap Motion (Ultraleap Limited, Bristol, England) offers hand-tracking technology that can be integrated with various VR systems, providing users with a more immersive and intuitive experience by enabling interaction solely through hand gestures and movements. While some VR headsets, such as the HTC Vive Pro Eye (HTC Corporation, Taoyuan City, Taiwan), incorporate eye-tracking technology to complement traditional input methods, others, like the Sony PlayStation VR (Sony Interactive Entertainment Inc., San Mateo, CA, USA), utilize the PlayStation Camera for limited controller-free interactions based on body movement tracking. These advancements in controller-free interactions contribute to enhancing immersion and the user experience in VR environments.

Additionally, the final product of the VR-SIM CARERS program could minimize the use of buttons to simplify the ergonomics of the controller. One easy solution is to apply the constraint-based approach to augment the controllers [15]. In this case, the designers can modify the controllers to reduce the number of buttons, thus constraining the number of choices that the users need to be concerned with. One way to achieve this is through 3D scanning and printing. For instance, the controllers could be scanned and custom-designed covers developed to hide the buttons not used in the VR-SIM CARERS applications. Such re-design using the constraint-based approach with a focus on simplification has the potential to significantly reduce the cognitive load associated with their use [19]. By streamlining features and functions, these controllers may become more intuitive and user-friendly, thus minimizing the mental effort required for task execution. Clear feedback mechanisms, such as visual cues as to which buttons are to be used, help users comprehend their actions more easily, further reducing cognitive processing demands. Additionally, aligning the controller's design with user abilities and task requirements ensures that users can use them more efficiently, leading to a decrease in cognitive load during task performance. Past research supports the effectiveness of constraint-based design in reducing cognitive load and enhancing usability. For example, a study by Pushpakumar et al. (2023) demonstrated that simplifying interfaces and providing clear feedback led to a significant reduction in cognitive load and improved performance in computer-based tasks. This highlights the importance of considering cognitive factors in tool design and the potential benefits of constraint-based approaches to optimizing user experiences. In the case of VR-SIM CARERS, streamlining the technical demands makes the user less likely to be confused or overwhelmed, allowing them to immerse themselves in the content entirely.

Lastly, providing additional training may also be a good approach. Literature suggests that training plays a crucial role in enhancing users' ability to effectively utilize complex tools [20]. Through structured learning experiences and hands-on practice, training programs increase users' familiarity with the features and functions of complex tools, empowering them to navigate interfaces confidently and perform tasks efficiently. Additionally, training facilitates skill development by providing targeted instruction and opportunities for practice, enabling users to master the necessary techniques and strategies for optimal tool usage. Importantly, training is not a one-time event but an ongoing process that supports continuous improvement and keeps users updated with new features or updates. Research supports the effectiveness of training for complex systems, as demonstrated in a meta-analysis by Salas et al. [20], which highlights the positive impact of training on users' performance and proficiency in utilizing complex tools. In our context, comprehensive orientation sessions and subsequent refreshment training sessions for first-time VR users can increase their comfort and confidence. The developers and implementers of the VR-SIM CARERS program should consider training as a part of the implementation process.

In summary, the collected data regarding participants’ mixed enjoyment and struggles with VR controllers has several implications for the VR-SIM CARERS program. Firstly, the feedback underscores the importance of a user-centered design approach. The incorporation of participant feedback and addressing the usability issues with the VR controllers will enhance the overall user experience and, in turn, the effectiveness of the VR-SIM CARERS program. It is also essential that the developers of the VR-SIM CARERS program recognize the varying levels of proficiency and comfort with VR systems and account for this properly. Supplementary training or additional resources specifically addressing the Meta Quest 2 VR system, including both the headset and controllers, can help users overcome initial challenges and improve either proficiency or comfort with the technology.

The study's limitations, such as its small sample size and focus solely on participants from the Durham Region in Ontario, may compromise the generalizability and reliability of the findings. This restriction could hinder the detection of nuanced user experience differences and limit the applicability of the results to broader CG populations. Moreover, the homogeneity of participants from a single region may restrict the diversity of perspectives, further diminishing their generalizability. To mitigate these limitations, future research should conduct larger-scale studies with more diverse participants, particularly within the CG community, to ensure broader representativeness. Additionally, longitudinal evaluations could offer valuable insights into how user experiences with VR technology evolve over time, providing a more comprehensive understanding of its impact.

## Conclusions

Integrating VR, notably Meta Quest 2, into the CARERS program for dementia CGs offers promise but faces challenges. Initial user testing showed mixed feedback, with some enjoying the experience while others struggled with usability, particularly the controllers. This highlights the need for user-centered design and potential controller improvements. Limited prior VR experience among participants underscores the importance of robust training resources. Suggestions for improvement include exploring alternative controllers and simplifying ergonomics. Despite valuable insights, limitations like a small sample size and demographic homogeneity exist. Future research should focus on larger, more diverse studies and longitudinal evaluations. Iterative refinement based on user feedback is crucial for optimizing the VR-SIM CARERS program for dementia CGs.
